# Novel PEGylated Lipid Nanoparticles Have a High Encapsulation Efficiency and Effectively Deliver MRTF-B siRNA in Conjunctival Fibroblasts

**DOI:** 10.3390/pharmaceutics13030382

**Published:** 2021-03-13

**Authors:** Amisha Sanghani, Konstantinos N. Kafetzis, Yusuke Sato, Salsabil Elboraie, Julia Fajardo-Sanchez, Hideyoshi Harashima, Aristides D. Tagalakis, Cynthia Yu-Wai-Man

**Affiliations:** 1 Faculty of Life Sciences & Medicine, King’s College London, London SE1 7EH, UK; amisha.sanghani19@imperial.ac.uk (A.S.); julia.fajardo-sanchez@kcl.ac.uk (J.F.-S.); 2 Department of Ophthalmology, St Thomas’ Hospital, London SE1 7EH, UK; 3 Department of Biology, Edge Hill University, Ormskirk L39 4QP, UK; kafetzik@edgehill.ac.uk (K.N.K.); Elborais@edgehill.ac.uk (S.E.); 4 Faculty of Pharmaceutical Sciences, Hokkaido University, Kita-12, Nishi-6, Kita-ku, Sapporo 060-0812, Japan; y_sato@pharm.hokudai.ac.jp (Y.S.); harasima@pharm.hokudai.ac.jp (H.H.)

**Keywords:** lipid nanoparticle, siRNA, targeting peptide, glaucoma, fibrosis

## Abstract

The master regulator of the fibrosis cascade is the myocardin-related transcription factor/serum response factor (MRTF/SRF) pathway, making it a key target for anti-fibrotic therapeutics. In the past, inhibitors and small interfering RNAs (siRNAs) targeting the *MRTF-B* gene have been deployed to counter fibrosis in the eye, with the latter showing promising results. However, the biggest challenge in implementing siRNA therapeutics is the method of delivery. In this study, we utilised the novel, pH-sensitive, cationic lipid CL4H6, which has previously demonstrated potent targeting of hepatocytes and endosomal escape, to safely and efficiently deliver an MRTF-B siRNA into human conjunctival fibroblasts. We prepared two lipid nanoparticle (LNP) formulations, incorporating targeting cleavable peptide cY in one of them, and measured their physicochemical properties and silencing effect in human conjunctival fibroblasts. Both proved to be non-cytotoxic at a concentration of 50 nM and effectively silenced the *MRTF-B* gene in vitro, with the targeting cleavable peptide not affecting the silencing efficiency [LNP with cY: 62.1% and 81.5% versus LNP without cY: 77.7% and 80.2%, at siRNA concentrations of 50 nM (*p* = 0.06) and 100 nM (*p* = 0.09), respectively]. On the other hand, the addition of the targeting cleavable peptide significantly increased the encapsulation efficiency of the LNPs from 92.5% to 99.3% (*p* = 0.0005). In a 3D fibroblast-populated collagen matrix model, both LNP formulations significantly decreased fibroblast contraction after a single transfection. We conclude that the novel PEGylated CL4H6-MRTF-B siRNA-loaded LNPs represent a promising therapeutic approach to prevent conjunctival fibrosis after glaucoma filtration surgery.

## 1. Introduction

Fibrosis, the pathological dysregulation of wound healing, represents one of the largest unmet needs in clinical medicine. In contrast with acute inflammatory reactions, which are short-lived, fibrosis is typically the result of chronic inflammation and is considered responsible for nearly 45% of all deaths in the developed world [1]. In the eye, it has been shown that fibrosis plays a major role in the long-term success rate of glaucoma filtration surgery [2]. According to the World Health Organization, glaucoma is the leading cause of irreversible blindness worldwide, affecting at present 76 million people and an estimated 112 million people by 2040 [3]. Currently, non-specific antimetabolite drugs, namely, mitomycin-C (MMC) and 5-fluorouracil (5-FU), are being used in glaucoma filtration surgery to modulate wound healing. They both act by suppressing DNA synthesis and causing non-specific apoptosis, but they are also known to cause serious vision-threatening complications, such as tissue damage and severe infections [4,5,6]. There is thus an unmet need for the development of novel, targeted and non-cytotoxic therapeutic agents.

The ubiquitous Serum Response Factor (SRF) and its co-activators, Myocardin-Related Transcription Factors A and B (MRTF-A and MRTF-B), are known master regulators of cytoskeletal gene expression [7,8]. Moreover, it has been shown that the MRTF/SRF pathway plays an important role in myofibroblast activation [9] and is intricately linked to most of the key regulators and pathways in fibrosis [10,11,12], making it a promising therapeutic target.

Small interfering RNAs (siRNAs) are short, non-coding, double-stranded RNA molecules, with an average length of 20–25 nucleotides that regulate genes and genomes at some of the most important levels of genome function, namely during transcription and translation [13,14]. The major advantage of the siRNA technology is that virtually any gene can be targeted and silenced by specifically degrading its mRNA, making it a powerful therapeutic tool in different diseases [15,16].

In the past, locally delivered MRTF/SRF inhibitors [17] and siRNA nanocomplexes [18,19] have been used to silence *MRTF-B* expression in fibroblasts and in animal models of glaucoma filtration surgery, showing promising results. One of the major hurdles for siRNA-based therapeutics is their delivery; therefore, in this study, we developed and optimised lipid nanoparticles (LNPs) to safely and effectively deliver MRTF siRNA into fibroblasts [20,21]. For their preparation, the novel, pH-sensitive and cationic lipid CL4H6 was used, in addition to 1,2-dioleoyl-sn-glycero-3-phosphoethanolamine (DOPE) and polyethylene glycol (PEG) lipids.

The CL4H6 lipid was identified and optimised after screening a pH-sensitive, cationic lipid library for lipids that could effectively deliver siRNAs into hepatocytes and induce a potent gene-silencing response [22,23]. Moreover, CL4H6 was shown to be biodegradable and well tolerated in vivo [22], and it displayed exceptional efficiency of endosomal escape and cytosolic release [22,23], which would also add to the efficiency of a therapeutic approach. In fact, it was proven that the CL4H6-LNP is superior to the efficiency of DLin-MC3-DMA [24,25], a lipid ingredient of Patisiran, which is the first siRNA-based drug that has been approved by the FDA for the treatment of polyneuropathy in patients with hereditary transthyretin-mediated amyloidosis (hATTR) [26], thus making it an excellent tool for our study.

In recent years, cell targeting peptides (CTPs) have been increasingly used with nanocarriers to achieve targeted delivery of drugs and other therapeutic agents with high specificity [27]. These CTPs are recognised by cell surface receptors, instigating the internalisation of their cargos, and thus allowing the delivery of therapeutics in specific cells. In this study, we used the cleavable version of the targeting peptide Y (cY). cY consists of a positively charged nucleic acid binding domain composed of 16 lysines (K_16_), a GA spacer, a targeting domain (CYGLPHK) and a cleavable RVRR linker [Arg-Val-Arg-Arg↓ (Arg = Arginine, Val = Valine, ↓ = indicating the cleavage site)] that is recognised by endosomal enzymes, such as cathepsin B and furin, which bind and cleave the peptide once inside the cells [28]. Peptide Y was identified by a phage peptide library for peptides that could bind to the cell surface receptors of airway epithelial cells with high affinity. The amino acid sequence of this peptide closely resembles part of a protein expressed by the pathogen *Legionella pneumophila* [29]. Although its specific target is still unknown, we have demonstrated that peptide Y mediates the targeted delivery of nanoparticles to a variety of tissues, including lung cells [30] and eye fibroblasts [18].

Here, we demonstrate for the first time that neutral PEGylated CL4H6 LNPs can efficiently deliver siRNAs into human conjunctival fibroblasts and achieve superior silencing efficiencies in vitro, while also displaying low cytotoxicity. Moreover, we evaluated for the first time the addition of a targeting peptide to these LNPs by biophysical characterisations and by performing transfections of human conjunctival fibroblasts to assess both gene silencing and functional efficiencies.

## 2. Materials and Methods

### 2.1. Materials

The pH-sensitive cationic lipid CL4H6 was made in house as previously described [22]. 1,2-Dioleoyl-sn-glycero-3-phosphoethanolamine (DOPE) and 1,2-dimirystoyl-sn-glycero, methoxyethylene glycol 2000 ether (PEG-DMG) were purchased from SIGMA Aldrich (St. Louis, MO, USA). Cleavable peptide cY (K16RVRRGACYGLPHKFCG) was synthesised by AMS Biotechnology (Abingdon, UK). The RiboGreen Assay kit was purchased from ThermoScientific (Loughborough, UK). MRTF-B siRNA and IRR (irrelevant control) siRNA were purchased from Horizon Discovery (Cambridge, UK), and the sequences for the sense and antisense strands of siRNAs are shown in Table 1.

### 2.2. Nanoparticle Formulations

CL4H6, DOPE and PEG-DMG were prepared in 90% t-BuOH at a final volume of 400 μL, a molar ratio of 50:50:1 and a final total lipid concentration of 1.4 mg/mL. Then, 100 μL of a 0.4 mg/mL siRNA solution were mixed with the lipids to give an N/P ratio of 7.5. Next, the LNPs were prepared in 20 mM MES buffer (pH 6.0) as previously described [22]. A portion of the resulting LNPs were mixed with peptide cY at a weight ratio of 4:1 (peptide:siRNA) by rapid mixing to obtain the LNP+cY nanoparticles. The LNPs were stored at 4 °C until needed.

### 2.3. Cell Culture

Human conjunctival fibroblasts were obtained from donor conjunctiva of glaucoma patients after informed consent and were cultured as previously described [18]. Fibroblasts between passages 2–8 were used in the experiments. All experimental protocols were approved by the West of Scotland Research Ethics Committee (REC 19/WS/0146).

### 2.4. Nanoparticle Size and Zeta Potential

A Nano ZS Zetasizer (Malvern Instruments, Malvern, UK) was used to measure the size and zeta potential of the nanoparticles by dynamic light scattering (DLS) and laser Doppler anemometry, respectively, as previously described [18]. Measurements were performed in triplicates for each sample and the results were analysed using the built-in software (DTS version 5.03).

### 2.5. Transmission Electron Microscopy

Nanoparticles were applied to a copper grid (300-mesh) coated with Formvar/carbon support film (Agar Scientific, Stanstead, UK). They were then incubated for 3 min at room temperature. The samples were negatively stained with uranyl acetate (1%) for 15 s, followed by air drying and blotting on filter paper. A Philips CM120 BioTwin TEM was used for imaging (accelerating voltage 120kV). The images were captured using an AMT 5MP digital TEM camera (Deben UK, Suffolk, UK).

### 2.6. Encapsulation Assay

The siRNA encapsulation efficiency was determined using the RiboGreen assay. 100 µL of nanoparticles or siRNA solution (for standard curve) were diluted in 100 µL of 10 mM HEPES buffer (pH 7.4) containing 20 µg/mL dextran sulfate and RiboGreen, in the presence or absence of 0.1 *w/v*% Triton X-100 (Sigma Aldrich, Gillingham, UK) added to a 96-well plate (Falcon, Fisher Scientific, Loughborough, UK). Fluorescence was measured using FLUOstar Omega (BMG LABTECH, Aylesbury, UK. The siRNA concentration was calculated based on an siRNA standard curve. The siRNA encapsulation efficiency was calculated by comparing the siRNA concentrations in the presence and absence of Triton X-100. The formula used was: siRNA encapsulation % = (Encapsulated siRNA-Unencapsulated siRNA)**/**(Total siRNA concentration) × 100.

### 2.7. In Vitro Transfection

Human conjunctival fibroblasts were seeded in 6-well plates (Falcon, Fisher Scientific, Loughborough, UK) at a density of 1 × 10^5^ cells per well and were incubated for 24 h to reach approximately 40% confluency before the LNPs were added. The cells were then incubated for 48 h at 37 °C with four types of LNPs in complete media: LNP-MRTF-B siRNA, LNP+cY-MRTF-B siRNA, LNP-IRR siRNA, LNP+cY-IRR siRNA. Each type of LNP was tested at two different siRNA concentrations (50 nM, 100 nM).

### 2.8. Real-Time Quantitative PCR

Human conjunctival fibroblasts were prepared for RNA extraction as per the manufacturer’s instructions using a RNeasy mini kit (Qiagen, Crawley, UK). The high-capacity cDNA reverse transcription kit was used for reverse transcription as per the manufacturer’s instructions (Applied Biosystems, UK). RT-qPCR reactions were performed on a ViiA7 Real-Time PCR system (Thermo Fisher Scientific, Loughborough, UK) using QuantiFast SYBR Green master mix (Qiagen, Crawley, UK). Assay conditions for RT-qPCR were as follows: stage 1, 95 °C for 5 min; stage 2, 95 °C for 10 s; then 60 °C for 30 s; repeated 45 times. The human primers were: MRTF-B, 5′-CTTCCTGTGGACTCCAGTG-3′, 3′-TGTGACTCCTGACTCGCAG-5′; GAPDH, 5′-ACGGATTTGGTCGTATTGGGC-3′, 3′-TTGACGGTGCCATGGAATTTG-5′. The mRNA values were normalised relative to those of GAPDH, and each experimental condition was carried out in triplicates.

### 2.9. Cell Proliferation Assay

Human conjunctival fibroblasts were seeded in 96-well plates at a density of 6.25 × 10^3^ cells per well (Falcon, Fisher Scientific, Loughborough, UK). Each nanoparticle formulation was added in triplicate wells for our measurements. Cell Titer 96 Aqueous one solution cell proliferation assay (Promega, Southampton, UK) was used to determine cell viability. The existing medium in each well was discarded and replaced with 100 μL of fresh growth medium, as well as 20 μL of the Cell Titer 96 Aqueous one solution. The plates were then incubated for 2 h at room temperature. Following this, the absorbance readings were recorded using a FLUOstar Omega (BMG LABTECH, Aylesbury, UK) at 540 nm. The cell viability was calculated as a percentage of the viability of the control untreated cells.

### 2.10. Collagen Contraction Assay

Human conjunctival fibroblasts were trypsinised and their cell density in the suspension was calculated. Cell suspensions with a cell density of 1 × 10^5^ cells/mL were centrifuged for 5 min at 1500 rpm. The supernatant was discarded and the cell pellet was resuspended in 100 µL of fetal calf serum. A collagen gel solution was prepared using 1 mL of Type 1 collagen at a concentration of 2.05 mg/mL in 0.6% acetic acid (First Link UK Ltd., Wolverhampton, UK), and 160 µL of concentrated medium [140 µL L-glutamine (ThermoScientific, Loughborough, UK), 1.4 mL DMEM 10X (Sigma Aldrich, Gillingham, UK) and 360 µL sodium bicarbonate 7.5% (Sigma Aldrich, Gillingham, UK)]. Before the cells were added to the solution, the pH was adjusted to pH 7.0 using sodium hydroxide (Sigma Aldrich, Gillingham, UK). Once the cells were added to the collagen gel solution, the fibroblast-gel mixture was placed in the MatTek dishes (MatTek Life Sciences, Ashland, MA, USA) and allowed to set for 10 min in an incubator (37 °C with 5% CO2 and 95% humidity). Each gel disc was gently detached from the edges of the well and 2 mL of growth medium was added before being placed back into the incubator. To measure matrix contraction, digital images were taken immediately following the release of the polymerised matrices (t_0_) and then daily over a period of 1 week (t_n_). Image J software (http://rsb.info.nih.gov/ij/, accessed on 5 February 2021) was used to analyse the images. The following formula was applied to calculate the gel surface area [A (t_n_) in % = 100–(100 × rt_n_2/rt_0_2)], where A is the gel surface area (this value was normalised to the value calculated at t_0_) and r is the radius. For each experimental condition, triplicate matrices were performed.

### 2.11. Statistical Analyses

To determine statistical significance, a Student’s t-test was performed and *p* values were calculated. ANOVA analysis was also used to determine statistically significant differences between the means of three or more treatment groups. Values that were statistically significant were expressed as follows: *, *p* < 0.05; **, *p* < 0.01; ***, *p* < 0.001. All graphs used in the paper display mean and standard error of the mean (SEM).

## 3. Results

### 3.1. Development and Biophysical Characterisation of Lipid Nanoparticles (LNPs)

**Figure 1:**
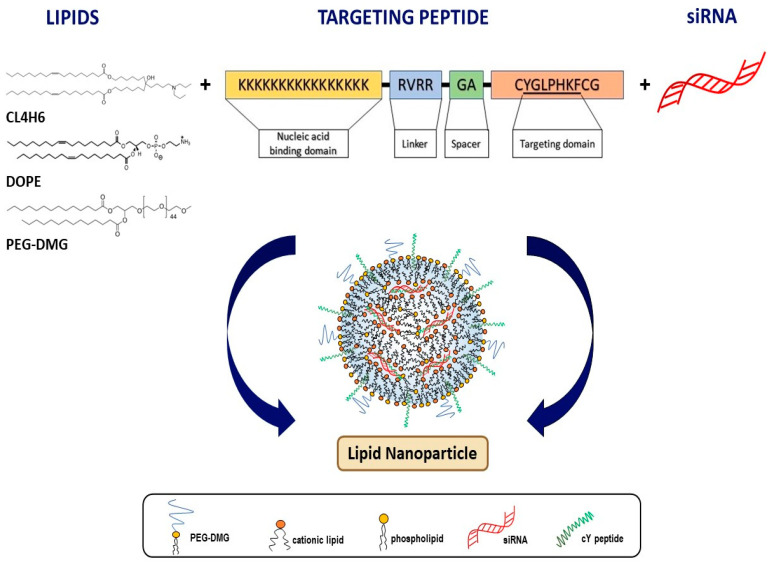
Schematic diagram of lipid nanoparticles with targeting cleavable peptide Y (cY).

We developed and characterised four LNP formulations: LNP-MRTF-B siRNA, LNP+cY-MRTF-B siRNA, LNP-IRR siRNA, LNP+cY-IRR siRNA (Figure 1).

**Figure 2:**
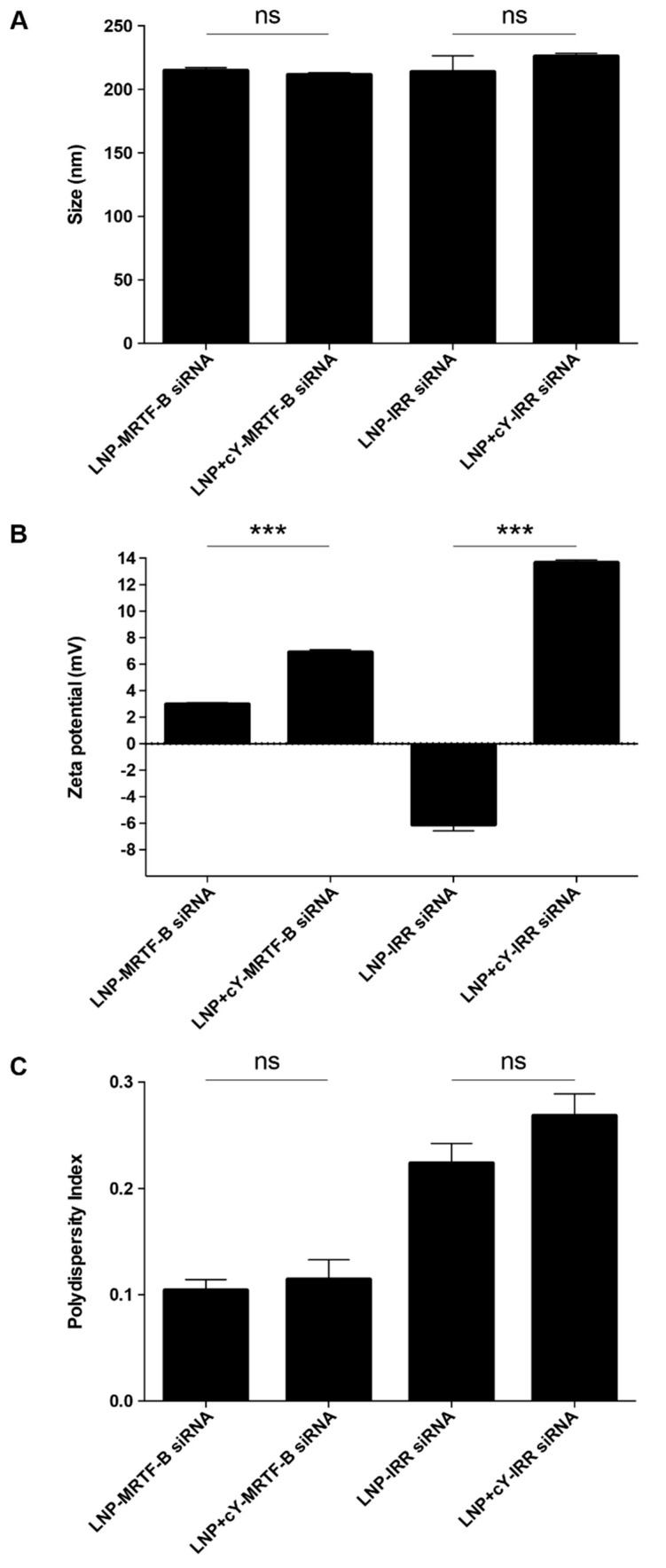
Biophysical characterisation of different lipid nanoparticle (LNP) siRNA formulations. (**A**) Size in nm. (**B**) Zeta potential in mV. (**C**) Polydispersity index. Results represent mean ± SEM. ***, *p* < 0.001; ns, not significant.

LNP-MRTF-B siRNA and LNP+cY-MRTF-B siRNA with cleavable peptide Y were similar in size, with a mean size of 214.4 ± 2.6 (SEM) nm and 211.2 ± 1.9 nm (*p* = 0.3749), respectively (Figure 2A). LNP-IRR siRNA and LNP+cY-IRR siRNA also had a similar mean size of 213.3 ± 12.9 nm and 225.8 ± 2.4 nm (*p* = 0.3978), respectively.

LNP-MRTF-B siRNA and LNP+cY-MRTF-B siRNA with cleavable peptide Y were weakly cationic and had a mean charge of +2.9 ± 0.1 mV and +6.9 ± 0.2 mV (*p* = 0001), respectively (Figure 2B). On the other hand, LNP-IRR siRNA were weakly anionic and had a mean charge of −6.1 ± 0.5 mV, whereas LNP+cY-IRR siRNA with cleavable peptide Y were cationic with a mean charge of +13.6 ± 0.2 mV (*p* = 0.000004). Therefore, all nanocomplexes were near neutral [31].

LNP-MRTF-B siRNA and LNP+cY-MRTF-B siRNA with cleavable peptide Y had low polydispersity indices (PDIs) of 0.10 ± 0.01 and 0.11 ± 0.02 (*p* = 0.6541), respectively, indicating a monodisperse population (Figure 2C). LNP-IRR siRNA and LNP+cY-IRR siRNA with cleavable peptide Y had higher PDIs of 0.22 ± 0.02 and 0.27 ± 0.02 (*p* = 0.1850), respectively.

**Figure 3:**
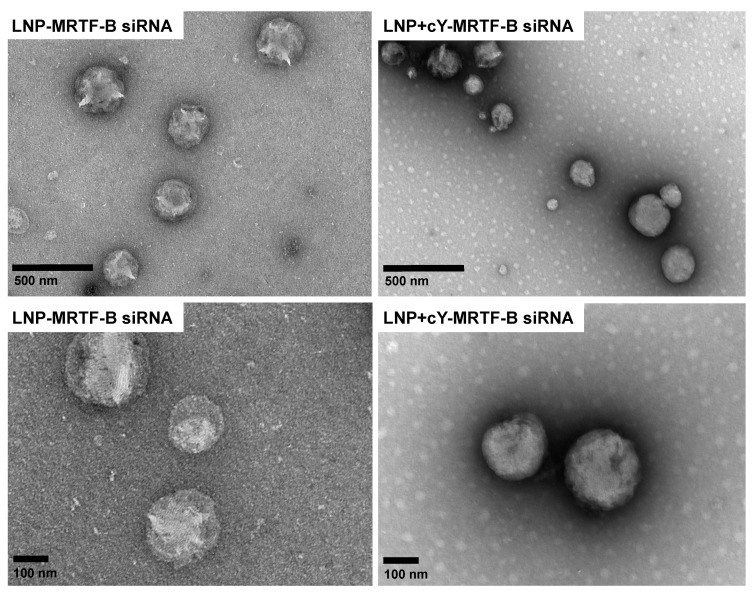
Negative staining transmission electron microscopy (TEM) imaging of LNP-MRTF-B siRNA (Left panel) and LNP+cY-MRTF-B siRNA with cleavable peptide Y (Right panel). The upper panels represent lower magnification (scale bar, 500 nm), while the lower panels represent higher magnification (scale bar, 100 nm).

LNP-MRTF-B siRNA and LNP+cY-MRTF-B siRNA with cleavable peptide Y were also characterised using negative staining TEM. The TEM imaging showed a spherical morphology in both LNP formulations and an approximate size of 200 nm (Figure 3), which is in accordance with the DLS measurements in Figure 2A.

### 3.2. Targeting Cleavable Peptide cY Significantly Increases Encapsulation Efficiency of LNPs

**Figure 4:**
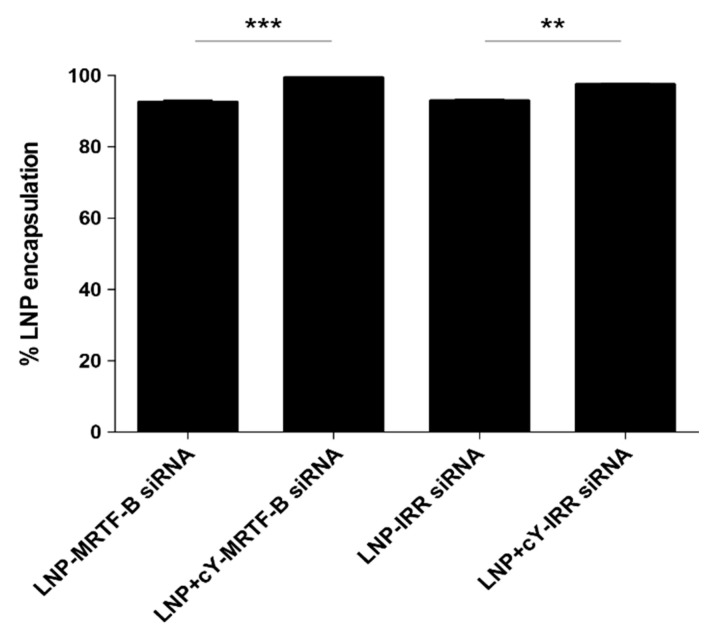
Percentage encapsulation efficiency of the LNP formulations: LNP-MRTF-B siRNA, LNP+cY-MRTF-B siRNA, LNP-IRR siRNA, LNP+cY-IRR siRNA. Results represent mean ± SEM for three independent replicates. **, *p* < 0.01; ***, *p* < 0.001.

The LNP-MRTF-B siRNA concentration was 40 ng/μL and after performing the encapsulation assay, it was calculated as 37.0 ng/μL. The LNP+cY-MRTF-B siRNA concentration with cleavable peptide Y was 39.4 ng/μL and after performing the encapsulation assay, it was calculated as 39.1 ng/μL. The percentage LNP encapsulation significantly increased from 92.5 ± 0.7% for LNP-MRTF-B siRNA to 99.3 ± 0.1% for LNP+cY-MRTF-B siRNA (*p* = 0.0005) (Figure 4).

The LNP-IRR siRNA concentration was 40 ng/μL and after performing the encapsulation assay, it was calculated as 37.1 ng/μL. The LNP+cY-MRTF-B siRNA concentration with cleavable peptide Y was 39.4 ng/μL and after performing the encapsulation assay, it was calculated as 38.3 ng/μL. The percentage LNP encapsulation also significantly increased from 92.8 ± 0.6% for LNP-IRR siRNA to 97.4 ± 0.4% for LNP+cY-IRR siRNA (*p* = 0.0032) (Figure 4).

### 3.3. LNPs Achieve Efficient Silencing of MRTF-B Gene Expression in Human Conjunctival Fibroblasts

**Figure 5:**
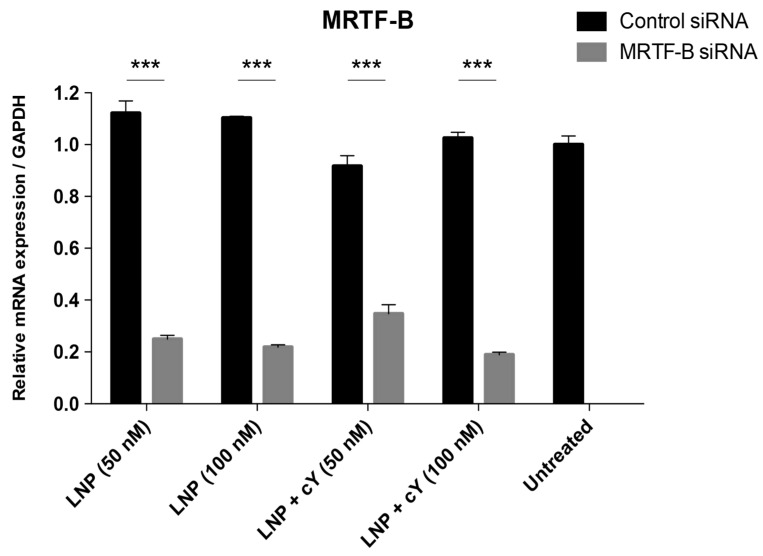
*MRTF-B* gene silencing in human conjunctival fibroblasts after transfection with LNP formulations with and without cleavable peptide Y, and at siRNA concentrations of 50 nM and 100 nM. mRNA levels were normalised relative to that of GAPDH and the results represent mean ± SEM for triplicate experiments. ***, *p* < 0.001.

Without cleavable peptide Y, the silencing efficiency of LNP-MRTF-B siRNA on *MRTF-B* gene expression was significantly increased in human conjunctival fibroblasts from 77.7% (*p* = 0.00006) to 80.2% (*p* = 0.0000002) at siRNA concentrations of 50 nM and 100 nM, respectively, compared to LNP-IRR siRNA (Figure 5).

With cleavable peptide Y, the silencing efficiency of LNP+cY-MRTF-B siRNA on *MRTF-B* gene expression was significantly increased in human conjunctival fibroblasts from 62.1% (*p* = 0.0005) to 81.5% (*p* = 0.000005) at siRNA concentrations of 50 nM and 100 nM, respectively, compared to LNP+cY-IRR siRNA (Figure 5).

The addition of cleavable peptide Y to LNP+cY-MRTF-B siRNA formulations did not have a significant effect on the silencing efficiency of *MRTF-B* gene expression in human conjunctival fibroblasts at siRNA concentrations of 50 nM (*p* = 0.06) and 100 nM (*p* = 0.09), compared to LNP-MRTF-B siRNA formulations.

### 3.4. LNPs Display Low Cytotoxicity at 50 nM siRNA Concentration in Human Conjunctival Fibroblasts

**Figure 6:**
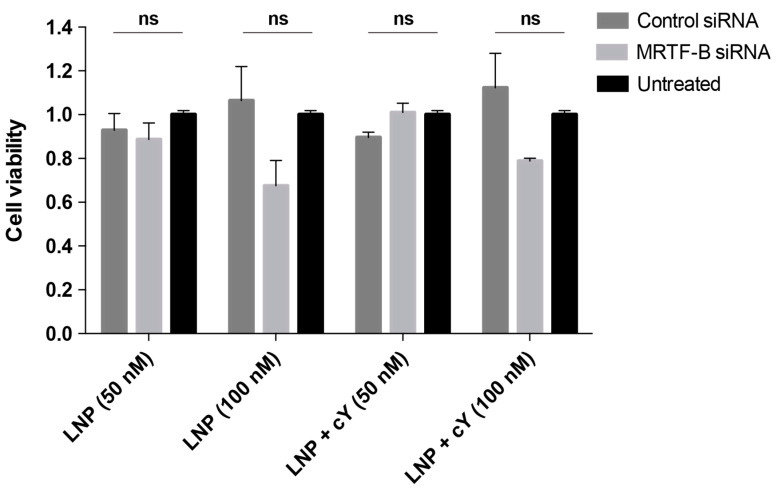
Cell viability of human conjunctival fibroblasts after transfection for 48 h with LNP formulations with and without cleavable peptide Y, and at siRNA concentrations of 50 nM and 100 nM. The siRNA concentrations at 50 nM and 100 nM were 0.693 μg/mL and 1.386 μg/mL, respectively. The total lipid amount at 50 nM was 25.2 μg for LNP+cY and 26.7 μg for LNP. At 100 nM, the total lipid amount was 50.2 μg for LNP+cY and 53.3 μg for LNP. Results represent mean ± SEM for three independent replicates. ns, not significant.

At a siRNA concentration of 50 nM, LNP-MRTF-B siRNA without cleavable peptide Y were not cytotoxic in human conjunctival fibroblasts compared to LNP-IRR siRNA or untreated control cells (F(2,6) = 0.83, *p* = 0.48) (Figure 6). At a siRNA concentration of 50 nM, LNP+cY-MRTF-B siRNA with cleavable peptide Y were also not cytotoxic in human conjunctival fibroblasts compared to LNP+cY-IRR siRNA or untreated control cells (F(2,6) = 4.22, *p* = 0.07).

At a siRNA concentration of 100 nM, LNP-MRTF-B siRNA without cleavable peptide Y showed lower cell viability compared to LNP-IRR siRNA or untreated control cells, but the difference was not statistically significant (F(2,6) = 3.44, *p* = 0.10) (Figure 6). At a siRNA concentration of 100 nM, LNP+cY-MRTF-B siRNA with cleavable peptide Y treatment also led to decreased cell viability compared to LNP+cY-IRR siRNA or untreated control cells, but the results were not statistically significant (F(2,6) = 3.38, *p* = 0.10).

### 3.5. LNP-MRTF-B siRNA Decrease Matrix Contraction after a Single Transfection Treatment

As LNP-MRTF-B siRNA and LNP+cY-MRTF-B siRNA formulations efficiently silenced *MRTF-B* gene expression and displayed low cytotoxicity at a siRNA concentration of 50 nM, we further tested the effect of these LNPs on fibroblast contractility using a functional collagen contraction assay. A three-dimensional free-floating fibroblast-populated collagen contraction model was used as it is a validated in vitro model of tissue contraction in the eye and includes two mechanisms: direct cell-mediated contractile activity and matrix degradation by the fibroblasts [32,33,34,35]. Drugs that have been shown to be effective in the three-dimensional free-floating fibroblast-populated collagen contraction model have also been found to be effective in the rabbit model of glaucoma filtration surgery [36,37].

**Figure 7:**
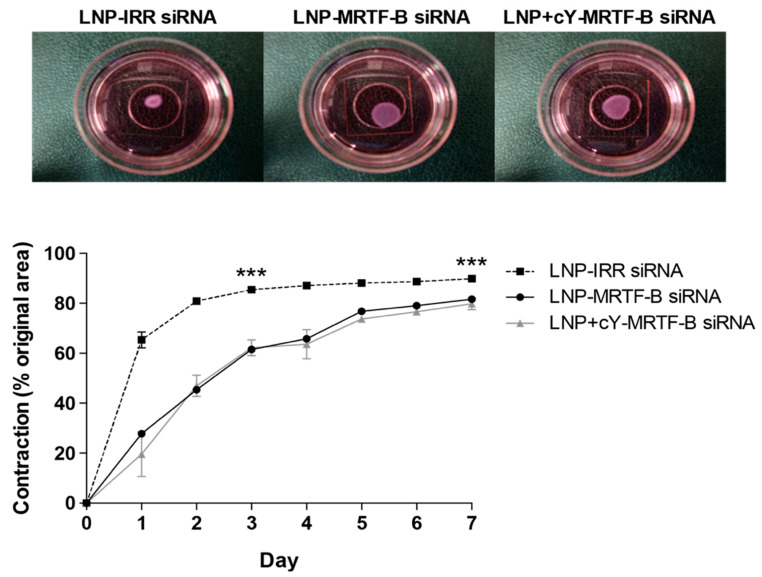
Three-dimensional fibroblast-populated collagen contraction assay of LNP-MRTF-B siRNA and LNP+cY-MRTF-B siRNA compared to LNP-IRR siRNA. All LNP formulations were tested at siRNA concentrations of 50 nM. Results represent mean ± SEM for three independent replicates. Representative gel areas at day 3 of contraction assay are shown. ***, *p* < 0.001.

Analysis of the data at day 3 showed that there was a significant relationship between matrix contraction and LNP siRNA treatment (F(2, 6) = 329.4, *p* < 0.0001). At day 3, LNP-MRTF-B siRNA and LNP+cY-MRTF-B siRNA decreased matrix contraction by 28.0% (*p* < 0.0001) and 27.2% (*p* < 0.0001), respectively, compared to LNP-IRR siRNA (Figure 7). There were no statistically significant differences in matrix contraction between LNP-MRTF-B siRNA and LNP+cY-MRTF-B siRNA at day 3 (*p* = 0.7856).

Analysis of the data at day 7 showed that there was a significant relationship between matrix contraction and LNP siRNA treatment (F(2,6) = 42.4, *p* = 0.0003). At day 7, LNP-MRTF-B siRNA and LNP+cY-MRTF-B siRNA decreased matrix contraction by 9.8% (*p* = 0.0007) and 10.7% (*p* = 0.0004), respectively, compared to LNP-IRR siRNA (Figure 7). There were no statistically significant differences in matrix contraction between LNP-MRTF-B siRNA and LNP+cY-MRTF-B siRNA at day 7 (*p* = 0.7825).

## 4. Discussion

Nanoparticle-mediated siRNA therapy presents us with many advantages over non-specific cytotoxic antimetabolite drugs, such as MMC, since it can offer greater cell specificity, potency and longer duration of the therapeutic effects [38,39]. Indeed, over the last decade, there has been a surge in the number of publications and clinical trials of novel siRNA-based therapeutics for ocular disorders [40,41,42,43,44,45].

We have previously used receptor-targeted nanocomplexes (RTNs), comprising oligolysine epithelial-targeting peptides and liposomes (e.g., DOTMA and DOPE), that self-assemble on mixing to form positively charged, monodisperse nanoparticles with an average diameter of approximately 100 nm, which can encapsulate and transfect siRNAs efficiently [46,47].

This study demonstrates that the PEGylated CL4H6-LNPs can effectively deliver the MRTF-B siRNAs into human conjunctival fibroblasts and lead to significant in vitro gene silencing of about 80%. Moreover, we have shown that the MRTF-B siRNA-loaded nanoparticles are not cytotoxic in vitro at a concentration of 50 nM. These pH-sensitive, near neutral PEGylated nanoparticles proved to have equally good silencing efficiency when compared to other targeted highly cationic non-PEGylated siRNA nanocomplexes that we have tested in the past [18,30], and they exhibited far superior silencing activity when compared to highly cationic PEGylated nanocomplexes [19,48]. This is possibly due to their ability to effectively escape from the endosomes via membrane fusion [22,23], as well as their high encapsulation efficiency. In addition, their near neutral charge might be another factor contributing to the effect of PEGylation in these formulations, which is in accordance with our other previous studies in which we showed that anionic PEGylated formulations efficiently silenced genes of interest [46]. Considering their high silencing efficiency, this would permit the use of even lower non-cytotoxic doses (<10 nM) in future studies.

By preparing LNPs with and without the targeting cleavable cY peptide, we wanted to test whether its addition had any effects on the physicochemical properties, cytotoxicity and transfection efficiency of the nanoparticles. Peptides with cleavable linkers, in this case an RVRR linker that is recognised by endosomal enzymes such as cathepsin B and furin, have been shown to transfect cells more efficiently compared to their non-cleavable peptide counterparts [28]. This is possibly due to the fact that once inside the endosomes, cleavage of the peptides can cause the nanoparticles to disengage from the receptor and be released from the endosomal membrane more efficiently. An important finding was that even though the addition of the cleavable cY peptide did not improve gene silencing, it did improve the encapsulation efficiency in our formulations to 99%, proving that the presence of the peptide forces the nanocomplexes to be tightly packed, protecting their cargo. An additional explanation could be that the positively charged RNA binding domain of the cY peptide can attract and bind multiple siRNA molecules in the interior of the LNPs; these electrostatic interactions prevent premature nucleic acid leakage from the nanoparticles, leading to increased encapsulation efficiencies [49,50].

Several studies have also reported a correlation between nanoparticle physicochemical properties (i.e., size, charge) and transfection efficiency, as well as cytotoxicity [51,52,53,54,55]. Furthermore, neutral nanoparticles are known to aggregate; however, the addition of PEG hinders LNP aggregation and reduces non-specific interactions by sterically precluding NPs from interacting with neighbouring NPs and blood/media components [56,57]. In addition, PEG modification increases the thickness of the fixed aqueous layer on the interface of LNPs, resulting in decreased zeta potential, which is the electrical potential at the slipping plane. In our study, the LNPs containing the MRTF-B siRNA, with and without cleavable peptide, were weakly cationic (+6.9 mV and +2.9 mV, respectively) with a size of around 200 nm, and a low polydispersity index (PDI) of around 0.1, indicating a homogenous nanoparticle population. On the other hand, the LNPs containing the control siRNA and cleavable cY peptide were more cationic (+13.6 mV), whereas the LNPs without the peptide were weakly anionic (−6.1mV). Moreover, they formed similar size nanoparticles (215 nm), with a slightly higher PDI (0.22 and 0.27, respectively), indicating a broader particle size distribution. Overall, the smaller, homogeneous nanocomplexes containing the MRTF-B siRNA were less or not cytotoxic to human conjunctival fibroblasts when compared to the control siRNA nanocomplexes.

Moreover, previous studies with freshly prepared cationic non-PEGylated nanocomplexes indicated that 70% *MRTF-B* gene silencing was sufficient to completely block matrix contraction in a seven-day collagen contraction assay [19]. Here, we were able to show a significant 30% decrease in matrix contraction with our PEGylated targeted and non-targeted LNPs at day 3. This difference might be attributed to the presence of PEG in our LNPs. Another contributing factor to the difference in these results was that our LNPs were stored for over 1 month in the fridge, whereas the other formulations used in our previous study were freshly made. Drug stability is a very important consideration when translating new therapeutics to patients in clinic and the LNPs demonstrated remarkable efficiency even when refrigerated for long periods, which makes them ideal as therapeutic nanoagents.

The next step is to test our formulations in vivo to verify their safety and efficiency. As shown in previous studies, nanoparticles can be locally administered to the glaucoma filtration surgery site [17,18]; thus future research would involve testing them in a pre-clinical animal model, such as the rabbit model of glaucoma filtration surgery [37,58,59]. The rabbit model is a validated and clinically relevant model for understanding and preventing tissue scarring. The agents that have shown a reduction in the formation of scar tissue in the rabbit model have also been shown to be effective in human clinical trials [60,61,62]. Future work should also focus on optimising the concentration, dosage and application of our therapeutic approach to achieve long-lasting effects that could prevent conjunctival fibrosis, since designing a drug that would only require a single administration at the time of the surgery remains a major challenge in glaucoma filtration surgery.

## Figures and Tables

**Table 1:** Structures of the different lipids and sequences of the peptides and small interfering RNAs (siRNAs).

Function	Lipids	Structure
Lipid	7-(4-(dipropylamino)butyl)-7-hydroxytridecane-1,13-diyl dioleate (CL4H6)	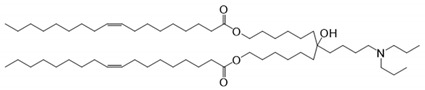
Lipid	1,2-dioleoyl-sn-glycero-3-phosphoethanolamine (DOPE)	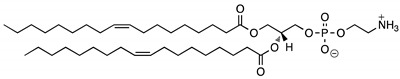
Lipid	1,2-dimirystoyl-sn-glycero, methoxyethyleneglycol 2000 ether (PEG-DMG)	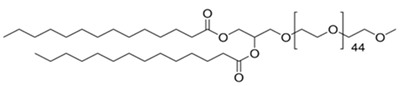
**Function**	**Peptide**	**Sequence**
Targeting	Cleavable Y (cY)	K_16_RVRR–GACYGLPHKFCG
**Function**	**siRNAs**	**Sequence**
Targeting	MRTF-B	Sense: GCA UCA UGC CAC CUU UGA AGA Antisense: UCU UCA AAG GUG GCA UGA UGC
Non-Targeting	Control IRR	Sense: UGG UUU ACA UGU CGA CUA A Antisense: UUA GUC GAC AUG UAA ACC A

## Data Availability

Not applicable.
